# Is there a relationship between parental concern and suspicion of developmental delay?

**DOI:** 10.1590/1984-0462/2025/43/2024187

**Published:** 2025-07-28

**Authors:** Mitali Lopes Melo, Daniela Akemi Tezuka Yamazaki, Luziani Leão dos Santos, Janaina Medeiros de Souza, Rafaela Silva Moreira

**Affiliations:** a Universidade Federal de Santa Catarina, Araranguá, SC, Brasil.; b Universidade Federal de Santa Catarina, Florianópolis, SC, Brasil.

**Keywords:** Child development, Child care, Parent-child relationships, Risk factors, Desenvolvimento infantil, Cuidado da criança, Relações pais-filho, Fatores de risco

## Abstract

**Objective:**

To investigate the existence of an association between parents’ concerns about their child’s development and alterations in the child’s development, risk factors, and family socioeconomic status.

**Methods:**

A cross-sectional study was performed, using data collected at child care centers in municipalities in the south of Santa Catarina, Brazil, from parents of children aged between four and 65 months. Information about parental concerns, child development, and family risk factors was collected using the "Survey of Wellbeing of Young Children (SWYC-BR)" instrument. The "Classification of the Brazilian Association of Research Companies" was applied to classify socioeconomic status. The data were then subjected to a multivariate analysis using the R Studio software. A significance level of 0.05 was adopted.

**Results:**

A total of 498 children participated in the study, with an average age of 41 months. The multivariate analysis revealed that parents who expressed concern about their children’s current development are approximately three times more likely to have children with suspected developmental delay (prevalence ratio – PR 2,97; 95% confidence interval – 95%CI 1.85–4.33). Furthermore, abuse of illicit substances increases the risk of children with suspected delay by almost two times (p=0.034). However, the other environmental variables were not associated with developmental delay.

**Conclusions:**

The study demonstrated that parents’ concern about their child’s development is a significant predictor of suspected developmental delay. This finding highlights the importance of considering parental concern as a factor in developmental monitoring and family-centered intervention programs.

## RESUMO

**Objetivo:**

Investigar a existência de associação entre a preocupação dos pais quanto ao desenvolvimento do filho com alterações no desenvolvimento da criança, fatores de risco familiares e condição socioeconômica.

**Métodos:**

Estudo transversal, no qual os dados foram coletados nos centros de educação infantil de municípios do sul de Santa Catarina, Brasil, com responsáveis por crianças de quatro a 65 meses. Questões sobre preocupação dos pais, desenvolvimento da criança e fatores de risco familiares foram levantadas por meio do instrumento "*Survey of Wellbeing of Young Children* (SWYC-BR)". Visando obter informações sobre a situação socioeconômica, foi aplicada a "Classificação da Associação Brasileira de Empresas de Pesquisa". Para a análise de dados, realizou-se uma análise multivariável utilizando o software R. Adotou-se nível de significância de 0,05.

**Resultados:**

Participaram do estudo 498 crianças com idade média de 41 meses. Encontrou-se, na análise multivariada, que os pais que expressaram preocupação com o desenvolvimento atual têm aproximadamente três vezes mais chances de os seus filhos apresentarem suspeita de atraso no desenvolvimento (razão de prevalência – RP 2,97; intervalo de confiança de 95% – IC95% 1.85–4.33). Além disso, o abuso de substâncias ilícitas apresentou associação significativa com a suspeita de atraso no desenvolvimento, enquanto as demais variáveis ambientais não foram associadas a este desfecho.

**Conclusões:**

O estudo demonstrou que a preocupação dos pais com o desenvolvimento de seus filhos apresentou-se como um preditor de suspeita de atraso no desenvolvimento. Esse achado destaca a importância de considerar a preocupação dos pais como um fator na vigilância do desenvolvimento e nos programas de intervenção centrados na família.

## INTRODUCTION

A recent survey conducted by the Ministry of Health in 13 Brazilian state capitals, involving 13 thousand Brazilian children, showed that approximately 12% of children up to the age of five years are suspected of having developmental delays, and that this incidence increases in families with greater social vulnerability.^1^ These data reinforce the need to closely monitor the children’s development in primary health care units. This developmental monitoring involves actions to promote typical development and early identification of possible abnormalities and is part of the primary health care practice for children. The monitoring of child development is recommended to be conducted by a multidisciplinary team comprising health professionals, family members or caregivers, and teachers, in accordance with the principle of shared responsibility.^1,2^

 In this monitoring process, parents’ concerns regarding their child’s development can have significant implications, serving as a guide for health professionals to make assessments and aiding decision-making.^3,4^ In the study by Glascoe et al., which included 971 American children aged zero to eight years, 73% of the parents had no development-related concerns about their children, indicating that these children presented age-appropriate development.^5^ Sheldrick et al. collected data from 451 American parents and found that the children of 87% of those reporting high levels of concern about their child’s development were positively screened for developmental delays during pediatric visits.^6^ These studies demonstrate that parental concern highly agrees with the results obtained by professionals using screening instruments. Therefore, parents can support healthcare professionals in clinical decision-making regarding development.^5,6^

 On the other hand, Cepanec et al. conducted a study with 211 Croatian children and demonstrated that 73% of them developed normally, despite parental concern.^7^ Reijneveld et al. gathered data from 4107 Dutch children and revealed that 12.5% of parents expressed concerns about a possible developmental delay. Nevertheless, 11.6% of the children for whom parents expressed concern did not demonstrate delays when assessed by health professionals.^8^ Both studies revealed a low agreement between parental concerns and health professionals’ assessments, indicating that parental concerns may not be a valid indicator for monitoring child development.^7,8^ Given that the association between parental concern and actual child’s development delay is not consistent across the literature, we sought to investigate the association between parental concerns about child development and actual developmental delays. 

 Besides, it is necessary to investigate whether environmental issues can influence the child’s development and, consequently, parental concerns. It is important to consider factors related to the environment and context in which the child is involved, as child development is multifactorial, resulting from the interaction of biological, environmental, family, and social factors.^9^ Food insecurity, family conflict, parental depression, alcoholism, drug use, and low family income are examples of risk factors that can interfere with child development. These factors, if present, can cause toxic stress for children and impact learning, behavior, and physical and mental health.^10^ Parents of children with multiple risk factors may experience heightened levels of concern, as financial constraints and unemployment can exacerbate these concerns.^11,12^

 The aim of this study was to examine the association between parents’ concerns about their children’s development and the occurrence of actual developmental delays in children aged zero to five years from the southern region of Santa Catarina, Brazil. In addition, the study sought to investigate the association of these actual developmental delays with risk factors in the family environment, such as food insecurity, family conflicts, parental depression, illicit substance abuse and the family’s socioeconomic level. This study has the potential to assist health professionals in incorporating parental concerns and parents’ perceptions regarding their children’s development into their assessments. 

## METHOD

 This is a cross-sectional analytical observational study that is part of a larger project approved by the Ethics Committee on Human Beings of the Federal University of Santa Catarina (Certificate of Presentation for Ethical Appreciation – CAAE: 68543917.1.0000.0121). 

 Data were collected from a convenience sample of public child care centers (CCC) in two municipalities in the extreme south of the state of Santa Catarina (SC). Araranguá (SC) has 71.922 inhabitants, 4589 of whom are children between the ages of zero and five. Its economy is based on agriculture, industry, and commerce. It exhibits a high infant mortality rate, with 16.11 deaths per one thousand live births. This is above the average for the state of Santa Catarina (9.8 deaths) and most Brazilian states.^13^ The city of Balneário Arroio do Silva (SC) has a population of 15.820, of whom 941 are children aged between zero and five. Its economy is based on services, industry, and agriculture.^13^

 The CCCs were selected by each city’s education departments, considering the number of children enrolled in the study age group, as well as those from regions of greater social vulnerability. Of the 18 CCCs in the municipality of Araranguá, eight were selected by the education department based on the above criteria, resulting in a convenience sample. In the municipality of Balneário Arroio do Silva, the education department selected one of the four existing CCCs. In 2023, Araranguá and Balneário Arroio do Silva had approximately 4 thousand children within the age group under study enrolled in public CCCs (3500 and 500 children, respectively). The study population consisted of parents/guardians and children without a previous medical diagnosis of neurological, cognitive, or physical disabilities, such as cerebral palsy or autism spectrum disorder, between the ages of four and 65 months. Parents/guardians were invited to participate when they picked up or dropped off their children at school and consented to participate in the study by signing the Informed Consent Form (ICF). 

 The study utilized two validated tools. The Survey of Wellbeing of Young Children (SWYC) is a North-American instrument that was culturally adapted for the Brazilian population in 2016.^14^ Since then, it has been used as a monitoring and screening tool to assess the development of children aged between one and 65 months.^15^ The SWYC-BR is divided into six questionnaires, three of which were implemented in this study: "Parent’s Concerns", "Developmental Milestones", and "Family Questions". 

 The "Parent’s Concerns" is a qualitative questionnaire intended to explore whether parents have any concerns regarding their children’s behavior and development. Responses are scored as "not at all", "somewhat", or "very much".^15,16^

 The "Developmental Milestones" questionnaire comprises ten questions to assess three domains: motor, cognitive, and language. The questionnaire contains 12 forms for different age ranges, tailored to cover children aged between one and 65 months. Response options range from zero to two, indicating the frequency with which the child performs the skills: 0 for "not at all", 1 for "a little", and 2 for "a lot". The total score is obtained by summing the responses from each item, with cut-off values for each age group indicating whether the child’s development is typical or not. If a child fails to meet the minimum score, professional assessment is recommended.^15^

 The "Family Questions" questionnaire assesses risk factors in the child’s family environment, including parental depression, family conflict, substance abuse, and food insecurity. The first four questions address the abuse of tobacco, alcohol and illicit substances. A positive screening occurs if any of these questions are answered affirmatively. The fifth question focuses on food insecurity, indicating a positive screening when respondents select "sometimes" or "always". Questions six and seven aim to identify maternal or paternal depression, with a score of three or above indicating a positive screening. Questions eight and nine assess the potential for family conflicts, with responses indicating possible domestic violence if "a lot of difficulty" and/or "a lot of conflict" are selected. Finally, question ten assesses the frequency of reading to children, on a scale of zero to seven, with higher scores indicating better practice.^15,16^

 The second tool employed was the Brazilian Association of Research Companies (ABEP) Classification, which is used to classify the socioeconomic status of the families using criteria such as purchasing power, access to public services, and the educational level of the head of the family. The Brazilian population is divided into six socio-economic strata, namely "A, B1, B2, C1, C2, and D-E", based on the total score obtained by summing all items.^17^

 The collection of data was executed subsequent to the approval from the education coordinators of each municipality and the directors of the CCCs, as well as parents/guardians, by means of the ICF. Data were collected by researchers who were previously trained, conducting face-to-face interviews, when parents/guardians were available, in the CCC teachers’ rooms using the SWYC-BR and ABEP questionnaires. Alternatively, when parents were unable to attend the CCCs, WhatsApp calls were conducted, with an average time of 15 minutes. The responses were documented on printed instruments. The outcomes were calculated to ensure that children exhibiting potential developmental delays were appropriately referred to healthcare teams for further evaluation based on their individual needs. Notwithstanding the recommendations of the Ministry of Health and the visits on childcare conducted in the municipalities evaluated, there is an absence of monitoring of child development in primary care and public CCCs. The purpose of this research is to contribute to reducing this gap in the existing services. 

 The information was entered into an Excel spreadsheet and a descriptive analysis was performed. The χ^2^ test was used to test the association between the scores of "change in developmental milestones" (dependent variable) and "parental concern" (independent and predictor variable), "abuse of illicit substance", "parental depression", "food insecurity", "domestic violence" and "socioeconomic level" (independent and adjusted variables). A prevalence ratio analysis was also performed in order to quantify the relationship between exposure factors (environmental factors) and the outcome (change in developmental milestones). 

 We also performed a multivariate analysis (logistic regression) and adopted a significance level of 0.05 in the final multivariate analysis model. For the regression analysis, the variable "change in developmental milestones" was defined as the outcome, and "parental concern", "illicit substance abuse", "food insecurity", and "socioeconomic status" as predictor variables. The variables "parental depression" and "domestic violence" were not included in the model due to the significant amount of missing data (above 20%). Quantitative data were entered, processed and analyzed using the R Studio software. 

## RESULTS

 Eight CCCs (57%) from the municipality of Araranguá and one (33.3%) from Balneário Arroio do Silva were evaluated. Of the 713 children enrolled in the nine CCCs under evaluation, 498 parents provided consent for their children to participate in the study. The study included 498 children, with an average age of 41.17 (±15.76) months, most of whom were female, and only 3.3% were preterm. Most families (57.83%) had the lowest socioeconomic levels (ABEP Levels C1, C2 and D-E). In these families, the father was the main contributor to the family income and had between 12 and 17 years of schooling (Table 1). 

**Table 1:** Sociodemographic characteristics of children and families.

Children’s characteristics	n=498	%
Sex		
Female	256	51.41
Gestational age*		
<37 weeks	14	3.26
Change in development milestones		
Yes	144	28.92
Family characteristics	n=498	%
Socioeconomic level		
A	13	2.61
B1	41	8.23
B2	156	31.33
C1	160	32.13
C2	101	20.28
D-E	27	5.42
Household head*		
Father	308	62.47
Mother	145	29.41
Other relatives	40	8.11
Years of schooling of head of household* (years)		
<12	148	31.23
≥12	326	68.78
Concern parental about the child’s development		
Yes	139	27.91

* Missing data.

 According to the Developmental Milestones questionnaire (SWYC-BR), 144 (28.92%) of the children evaluated presented suspected alterations in their overall development. Among the parents who participated in the study, 139 (27.91%) reported that they were currently concerned about their child’s development (Table 1). 

Table 2 presents the results of the univariate analysis, which was conducted to test the association between "change in development milestones" and the variables "parental concern about their children’s development", "abuse of illicit substances", "parental depression", "food insecurity", "domestic violence" and "socioeconomic level". The variable of parental concern yielded a significant p-value when associated with suspected developmental delay. In addition, abuse of illicit substances showed a statistically significant association with suspected developmental delay. However, the other environmental variables were not associated with the outcome suspected developmental delay (Table 2). 

**Table 2:** Association of changes in developmental milestones and parents’ concern and environmental variables.

Variables	Change in development milestones		Total (%)	p-value
	Yes n (%)	No n (%)		
Parental concerns				
Yes	64 (46.0)	75 (54.0)	139 (100)	**<0.001**
No	80 (22.3)	279 (77.7)	359 (100)	
Abuse of illicit substances				
Yes	45 (38.8)	71 (61.2)	116 (100)	**0.010**
No	99 (25.9)	283 (74.1)	382 (100)	
Parental depression*				
Yes	15 (38.5)	24 (61.5)	39 (100)	0.114
No	90 (27.7)	235 (72.3)	325 (100)	
Food insecurity*				
Yes	17 (37.0)	29 (63.0)	46 (100)	0.140
No	127 (28.2)	324 (71.8)	451 (100)	
Domestic violence*				
Yes	6 (50.0)	6 (50.0)	12 (100)	0.129
No	98 (26.0)	279 (74.0)	377 (100)	
Socioeconomic level				
High (A, B1 e B2)	58 (27.6)	152 (72.4)	210 (100)	0.329
Low (C1, C2 e D)	86 (29.9)	202 (70.1)	288 (100)	

* Missing data.

Bold indicates p-values<0.05.

Table 3 presents the final model resulting from the multivariate analysis and prevalence ratio analysis. The results indicate that parents who express concern about their children’s current development are approximately three times more likely to have children with suspected developmental delay (p<0.001). Furthermore, abuse of illicit substances increases the risks of children with suspected delay by almost two times (p=0.034). 

**Table 3:** Final model resulting from the multivariate analysis.

Variables	Prevalence ratio	Exp{B}	95%CI	p-value
Parental concerns	2.976	2.831	1.851–4.337	**<0.001**
Abuse of illicit substances	1.497	1.643	1.033–2.595	**0.034**
Food insecurity	1.389	1.011	0.503–1.970	0.974
Socioeconomic level	1.081	1.045	0.690–1.585	0.834

CI: confidence interval.

Bold indicates p-values<0.05.

## DISCUSSION

 The present study demonstrated an association between parental concern and actual developmental delays in their children, and also between the abuse of illicit substances and developmental delays. However, the study did not find an association between other risk factors present within the family environment, socio-economic status, and current development. 

 We found an association between parental concern and the suspicion of delays in children’s development. This finding is similar to that of Chung et al., who compared parental concern about motor development delays in 273 Chinese children under seven years old.^18^ The authors showed that 88% of parents who reported concerns about development had children with delays, while 80% of parents without concern had children without delays.^18^ Another research, conducted by Chen et al., included 101 Chinese children with an average age of 38.7 months. The study found that parental concerns had a high sensitivity (77–89%) when it came to diagnosing delays in the motor and language domains. Furthermore, the researchers discovered that parental concerns about global, motor, and language delays exhibited a high positive predictive value (55–77%), whereas concerns about cognition and behavior demonstrated relatively low positive predictive values (25–33%). The authors conclude that these findings suggest that such concerns should be carefully analyzed and that professionals should investigate other domains of these children’s development.^19^

 Different studies have also confirmed these results, emphasizing the significance of integrating parental screening in healthcare and pre-school settings.^20-22^ This integration is justified because parental concerns are close to the results of screening tests performed by health professionals and can be used to guide referral decisions.^20,21^ The analysis also indicated that children experienced an average absence of three and a half years from developmental monitoring. Thus, the present study revealed a deficiency in the integration of health and education services with parents in the municipalities evaluated. It is possible that routine pediatric appointments, available in basic health units, were predominantly centered on growth monitoring. In general, there is a lack of systematic evaluation of child development or consideration of the perspectives of parents and/or caregivers in primary health care units in Brazil. It is important to consider that the identification of developmental delays is a multifaceted process that engages numerous individuals who interact with the child on a daily basis. The findings of this study can contribute to the recognition and appreciation of the role of parents in this process, a role that has frequently been overlooked. 

 A scoping review was conducted to elucidate the mechanisms that facilitate parents’ perception of their children’s development.^3^ The review revealed that concerns are perceived through two mechanisms: knowledge of child development and comparison with other children. Notwithstanding the low socio-economic and educational level of the sample evaluated, which could influence these mechanisms, high levels of suspicion of developmental alterations and parental concern about the children’s development were obtained.^3^ The prevailing hypothesis is that the comparison with the development of other children in the same age group and living in the same context is the primary factor that arouses parents’ perception of possible alterations in development. 

 Further, an association was identified between illicit substance abuse and suspected developmental delay. In their research on Brazilian families, Delgado et al. demonstrated that exposure to tobacco smoke in the home can result in passive inhalation, which not only harms the child’s health but also leads to frequent hospitalizations for respiratory issues. This causes fear in the child and distances them from the school and family environment. Research conducted by Martikainen et al. indicated that children exposed to parental alcoholism are at an increased risk of developing psychiatric issues.^23^ Furthermore, Campelo et al. reported that Brazilian children with drug-addicted parents demonstrate impulsive behavior, inattention, low academic achievement, anxiety, and depression compared to non-exposed children.^24^ It is believed that parents are aware that these habits do not create a positive environment for their child’s full development, reflecting a growing concern. 

 The present study found no association between food insecurity and suspected developmental delay. In contrast to our findings, Rose-Jacobs et al. conducted a study in medical centers in the United States involving 2010 participants and demonstrated that caregivers experiencing food insecurity (n=427) were 67% more likely to report developmental risks in their children. The authors argue that food-insecure families are more prone to anxiety and daily stress due to the uncertainty of not having a secure food source. In our study, we believe that the greater socioeconomic vulnerability of the sample (presence of multiple environmental risk factors) when compared to high-income countries may have contributed to parents not perceiving risk to their children’s development.^25^

 The current study also did not find any association between parental depression and suspected developmental delay. However, maternal mental health represents a significant risk factor for child development, as depressed mothers may be less likely to create stimulating environments for their children compared to healthy mothers.^26^ In addition, Woolfenden et al. found in a systematic review that parental depression increases parental concern about the risk of developmental delay.^12^ The disagreement with the literature may be attributed to the small sample size of families with parental depression in comparison to those without. As a result, obtaining a statistically significant result was not possible. 

 The current study also found no association between domestic violence and suspected developmental delay, possibly due to the small number of reported incidents of violence. Tough et al. suggest that, among 791 Canadian mothers, 11% had significant concerns regarding their children’s development. Of these, 47% had experienced abuse prior to pregnancy, be it physical, emotional, or sexual.^27^ It is well documented that children born into adverse circumstances, where poverty and stress limit possibilities and aspirations, can experience adverse effects on their development.^28^ However, no articles were found concerning the relationship between parental concern and domestic violence in households where children are raised. 

 Furthermore, the children from low-income families in this study had no suspicion of developmental delay. A low family income can cause stress, affect parents’ self-esteem and sense of control, and impact the financial and material resources required to provide adequate care for a child.^26^ This result contradicts the findings of Sheldrick et al., who reported that 24% of 465 American parents expressed concern regarding their child’s development, with households earning less than $50 thousand dollars annually being more likely to report these concerns.^6^ Other studies with similar findings show an association between low income and a greater tendency to express concern about their child’s development.^8,12^ The homogeneity of the data between low and high socioeconomic status may have contributed to the non-significant results found. 

 To the best of the authors knowledge, this is the first Brazilian research study to examine parental concerns regarding child development. In addition, this study included a substantial number of participants, enabling us to demonstrate significant results for the research objectives. Additionally, both questionnaires employed were easy and quick to administer. However, it is necessary to acknowledge the limitation that the data on environmental variables were collected through screening instruments reported by the parents themselves, which include personal themes that may cause some embarrassment. This may have directly impacted the results of the family risk factors, as only a few positive screening reports were obtained, potentially compromising the associations with suspected developmental delay. It is important to elucidate that the children with a positive screening were not subjected to a subsequent follow-up to determine the accuracy of the developmental delay. In addition, because this was a convenience sample, the results of this study cannot be generalized. 

 In conclusion, parental concern about children’s development was identified as a predictor of suspected developmental delays. This finding highlights the importance of considering parental concern as a factor in developmental monitoring and family-centered intervention programs. Among the environmental factors examined, only illicit substance abuse was found to have an impact on the development of the children assessed. 

## Data Availability

The database that originated the article is available with the corresponding author.

## References

[1] Brazil (2023). Ministério da Saúde [homepage on the Internet]. Resumo Executivo – Projeto PIPAS 2022: Indicadores de desenvolvimento infantil integral nas capitais brasileiras.

[2] Caminha MFC, Silva SL, Lima MC, Azevedo PT, Figueira MC, Batista M (2017). Vigilância do desenvolvimento infantil: análise da situação brasileira. Rev Paul Pediatr.

[3] Cuomo B, Joosten A, Vaz S (2019). Scoping review on noticing concerns in child development: a missing piece in the early intervention puzzle. Disabil Rehabil.

[4] McLeod S, Crowe K, McCormack J, White P, Wren Y, Baker E (2018). Preschool children’s communication, motor and social development: parents’ and educators’ concerns. Int J Speech Lang Pathol.

[5] Glascoe FP (1999). Using parents’ concerns to detect and address developmental and behavioral problems. J Spec Pediatr Nurs.

[6] Sheldrick RC, Neger EN, Perrin EC (2012). Concerns about development, behavior & learning among parents seeking pediatric care. J Dev Behav Pediatr.

[7] Cepanec M, Lice K, ŠimleŠa S (2012). Mother-father differences in screening for developmental delay in infants and toddlers. J Commun Disord.

[8] Reijneveld SA, de Meer G, Wiefferink CH, Crone MR (2008). Parents’ concerns about children are highly prevalent but often not confirmed by child doctors and nurses. BMC Public Health.

[9] Pereira L, Guedes SC, Morais RL, Nobre JN, Santos JN (2021). Environmental resources, types of toys, and family practices that enhance child cognitive development. Codas.

[10] Sociedade Beneficente Israelita Brasileira Albert Einstein (2021). Nota técnica para organização da rede de atenção à saúde com foco na atenção primúria à saúde e na atenção ambulatorial especializada – saúde da criança.

[11] Algarvio S, Leal I, Maroco J (2012). Preocupações parentais em pais de crianças do pré-ecolar e 1o ciclo: análise da poupulação portuguesa por distrito. Psychology Community & Health.

[12] Woolfenden S, Eapen V, Williams K, Hayen A, Spencer N, Kemp L (2014). A systematic review of the prevalence of parental concerns measured by the Parents’ Evaluation of Developmental Status (PEDS) indicating developmental risk. BMC Pediatr.

[13] Brazil (2022). Instituto Brasileiro de Geografia e Estatística [homepage on the Internet].

[14] Moreira RS, Magalhães LC, Siqueira CM, Alves CR (2019). Cross-cultural adaptation of the child development surveillance instrument "Survey of Wellbeing of Young Children (SWYC)" in the Brazilian context. J Hum Growth Dev.

[15] Alves CR, Guimarães MA, Moreira RS (2022). Survey of Well-being of Young Children (SWYC-BR): manual de aplicação e interpretação.

[16] Perrin EC, Sheldrick C, Visco Z, Mattern K (2016). The Survey of Well-being of Young Children (SWYC) User’s Manual. 1.01.

[17] Associação Brasileira de Empresas de Pesquisa (2018). Critério Brasil e Alterações na aplicação do Critério Brasil, válidas a partir de 16/04/2018.

[18] Chung CY, Liu WY, Chang CJ, Chen CL, Tang SF, Wong AM (2011). The relationship between parental concerns and final diagnosis in children with developmental delay. J Child Neurol.

[19] Chen IC, Lee HC, Yeh GC, Lai CH, Chen SC (2004). The relationship between parental concerns and professional assessment in developmental delay in infants and children--a hospital-based study. J Chin Med Assoc.

[20] Glascoe FP (1997). Parents’ concerns about children’s development: prescreening technique or screening test?. Pediatrics.

[21] Ilić SB, Nikolić SJ, Ilić-Stošović DD, Golubović ŠS (2020). Early identification of children with developmental delay and behavioural problems according to parents concerns in the Republic of Serbia. Early Child Dev Care.

[22] Bjelland I, Posserud MB, Wergeland GJ (2022). Dialogue based early detection-development of a novel approach for detection of mental health problems among children in daycare centers. Front Psychiatry.

[23] Martikainen P, Korhonen K, Moustgaard H, Aaltonen M, Remes H (2018). Substance abuse in parents and subsequent risk of offspring psychiatric morbidity in late adolescence and early adulthood: a longitudinal analysis of siblings and their parents. Soc Sci Med.

[24] Campelo LL, Santos RC, Angelo M, Nóbrega MP (2018). Efeitos do consumo de drogas parental no desenvolvimento e saúde mental da criança. SMAD Rev Eletrônica Saúde Ment Álcool Drog.

[25] Rose-Jacobs R, Black MM, Casey PH, Cook JT, Cutts DB, Chilton M (2008). Household food insecurity: associations with at-risk infant and toddler development. Pediatrics.

[26] Stevens GD (2006). Gradients in the health status and developmental risks of young children: the combined influences of multiple social risk factors. Matern Child Health J.

[27] Tough SC, Siever JE, Leew S, Johnston DW, Benzies K, Clark D (2008). Maternal mental health predicts risk of developmental problems at 3 years of age: follow up of a community based trial. BMC Pregnancy Childbirth.

[28] Batista CL, Brentani AV (2023). Análise da influência do momento do ingresso em creches no desenvolvimento infantil. Cad Saúde Pública.

